# Quantum loop states in spin-orbital models on the honeycomb lattice

**DOI:** 10.1038/s41467-021-23033-y

**Published:** 2021-05-21

**Authors:** Lucile Savary

**Affiliations:** 1 Department of Physics, Massachusetts Institute of Technology, Cambridge, MA USA; 2 Université de Lyon, École Normale Supérieure de Lyon, Université Claude Bernard Lyon I, CNRS, Laboratoire de physique, Lyon, France

**Keywords:** Electronic properties and materials, Magnetic properties and materials

## Abstract

The search for truly quantum phases of matter is a center piece of modern research in condensed matter physics. Quantum spin liquids, which host large amounts of entanglement—an entirely quantum feature where one part of a system cannot be measured without modifying the rest—are exemplars of such phases. Here, we devise a realistic model which relies upon the well-known Haldane chain phase, i.e. the phase of spin-1 chains which host fractional excitations at their ends, akin to the hallmark excitations of quantum spin liquids. We tune our model to exactly soluble points, and find that the ground state realizes Haldane chains whose physical supports fluctuate, realizing both quantum spin liquid like and symmetry-protected topological phases. Crucially, this model is expected to describe actual materials, and we provide a detailed set of material-specific constraints which may be readily used for an experimental realization.

## Abstract (web summary)

Some one-dimensional chains host fractional excitations at their ends, akin to the hallmark excitations of quantum spin liquids. Here, Savary proposes a realistic model which uses such one-dimensional chains as building blocks for higher-dimensional exotic fluctuating quantum phases.

## INTRODUCTION

The original proposal of Anderson for quantum spin liquids (QSLs) involved resonating valence bonds, i.e. coherent superpositions of singlet coverings of the lattice^1–3^. More recently, proposals for both QSLs^4–6^ and symmetry-protected topological (SPT) phases^7–10^ have emerged, which are now based on fluctuating chains rather than singlets. More precisely, the building blocks are Haldane-like chains^11–13^, which are featureless in their bulk but host-protected gapless states confined to their ends. This is clearest in the AKLT chain (a representative state of the Haldane phase)^13^, where each spin one is rewritten as two spin half’s subsequently projected back onto the *S* = 1 representation at each site, and singlets form astride each bond. In this picture, two free *S* = 1/2 are indeed left at each end of open chains. The Haldane states are themselves one-dimensional SPTs; in the two- and three-dimensional Haldane-based QSL and SPT constructions, their physical supports fluctuate and their ends act as the bulk or edge fractional excitations, respectively, which characterize such phases. While such wavefunctions and even parent Hamiltonians have been proposed, it has remained far from obvious how they could be achieved in a realistic setting, let alone an actual material.

Independently, concrete spin-orbital (Kugel–Khomskii^14,15^) models, which capture single-site spin and orbital degeneracies, have been shown to host a rich spectrum of phenomena^15^, notably, valence bond solids^16–24^ and orbital liquids (e.g. refs. ^22,25–29^ and refs. ^30–33^ after this paper first appeared). The crucial ingredient is the modulation of the effective spin-exchange strength, which allows for stronger and weaker bonds to form, owing to the relationship between effective exchange strength and orbital overlap.

**Fig. 1:**
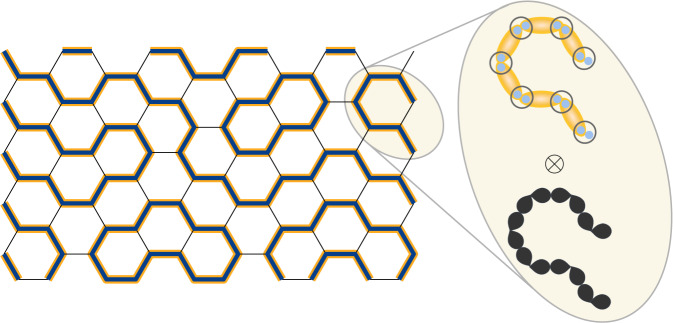
Pictorial representations of a (static) loop and orbital configuration. An orbital and Haldane loop covering a section of the honeycomb lattice.

Here, we show that orbital degrees of freedom provide a simple loop-forming mechanism, and allow to naturally realize the AKLT chain ground state picture (see Fig. 1). Specifically, we construct a spin-orbital model, i.e. a model with orbital degeneracy, on the honeycomb lattice for *S* = 1 and effective *L* = 1, which supports fluctuating Haldane chains (subtended by “orbital loops”, i.e. closed strings of bonds with large orbital overlap), a Haldane chain-based SPT, as well as a hexagon Haldane loop crystal with “Haldane-gap wave” excitations. When taken to the three-dimensional hyperhoneycomb lattice, the model is also home to a fully-fledged symmetry-enriched *U*(1) Coulombic spin-orbital liquid and a fractionalized antiferromagnet.

## RESULTS

We proceed as follows. We first introduce the appropriate ingredients and mathematical formalism and derive the minimal realistic model that induces the formation of fluctuating loops. Then, we analyze in detail the pure orbital part of the Hamiltonian and show how orbital loops emerge, before introducing spin degrees of freedom. The addition of a large spin exchange produces new, fluctuating, decorated loops. Along the way, we derive results in a large portion of the phase diagram we set out to study.

**Fig. 2:**
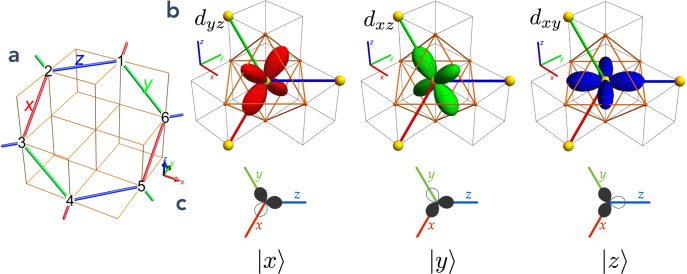
Pictorial representation of the orbital states. **a** The honeycomb lattice embedded in a cubic structure. Honeycomb planes are perpendicular to 〈111〉 axes, here the $[1\,\overline{1}\,1]$ axis. **b** The *t*_2*g*_ orbitals shown in a cubic environment, surrounded by a putative octahedral cage. **c** Pictorial representation of the $\left|x\right\rangle ,\left|y\right\rangle ,\left|z\right\rangle$ states. In state $\left|x\right\rangle$, the *d*_*yz*_ orbital is empty, while orbitals *d*_*xy*_ and *d*_*xz*_ each contain one electron. For clarity, only the lobes in the bond directions are shown.

We consider two electrons at each site of a honeycomb lattice, in degenerate *t*_2*g*_ = {*d*_*yz*_, *d*_*xz*_, *d*_*xy*_} orbitals (which we also denote for convenience *x*, *y* and *z* orbitals, respectively), with all other orbitals filled or empty and far away in energy from the *t*_2*g*_ manifold. We assume large Hund’s coupling *J*_H_, which enforces the high-spin state *S* = 1, and large intra-orbital repulsion *U*, which imposes no more than one electron per orbital. There are then two occupied and one empty orbital at each site, and the site Hilbert space is ${\mathcal{H}}={{\mathcal{H}}}_{{L}_{{\rm{eff}}} = 1}\times {{\mathcal{H}}}_{S = 1}$. The orbital-space basis $(|x\rangle ,|y\rangle ,|z\rangle )$ is defined such that in state $\left|x\right\rangle$ the *x* orbital is empty, while the other two are filled, and similarly for $|y\rangle$ and $\left|z\right\rangle$ (see Fig. 2b, c). [More formally, we can write that state $\left|\mu \right\rangle$ is such that *d*_*μ* − 1,*μ* + 1_ is empty, where *x* ± 1 = *y*, *z*, etc.]^34^. A set of nine operators acting in this space can be chosen to be {*L*^*μ*^, *P*^*μ*^, *T*^*μ*^} with *μ* = *x*, *y*, *z*, such that ${L}^{x}=i(|z\rangle \langle y|-|y\rangle \langle z|)$, ${P}^{x}=1-|x\rangle \langle x|={\hat{n}}^{x}$ and ${T}^{x}=-(|z\rangle \langle y|+|y\rangle \langle z|)$ and cyclic permutations. The *L*^*μ*^ operators are Hermitian, obey the angular momentum algebra and are such that ${L}^{\mu }|\mu \rangle =0$. *P*^*μ*^ is a projection operator that measures the occupation of the *μ* [i.e. *d*_*μ* − 1,*μ* + 1_ with (*x* ± 1 = *y*, *z* and permutations)] orbital, so that the two-electron per-site constraint is written ∑_*μ*_*P*^*μ*^ = *L*(*L* + 1) = 2. Moreover, [*P*^*μ*^, *P*^*ν*^] = 0.

We now write the minimal physically realistic Hamiltonian acting in ${\mathcal{H}}$, including only nearest-neighbor interactions, which realizes a resonating chain regime. We assume isotropy in spin space (no spin–orbit coupling) and a local cubic environment (necessary for *t*_2*g*_ orbitals). This Hamiltonian is

1 $$H= \, 	\mathop{\sum}\limits_{\langle ij\rangle }\left({P}_{i}^{{\gamma }_{ij}}{P}_{j}^{{\gamma }_{ij}}\left[-\zeta +J\left({{\bf{S}}}_{i}\cdot {{\bf{S}}}_{j}+\beta {({{\bf{S}}}_{i}\cdot {{\bf{S}}}_{j})}^{2}\right)\right]\right.\\ 	 -\left.\upsilon \left[{T}_{i}^{{\gamma }_{ij}-1}{T}_{j}^{{\gamma }_{ij}+1}+{\rm{h.c.}}\right]\right).$$

Except where otherwise noted, we take *ζ*, *J* ≥ 0 and −1 ≤ *β* ≤ 1. *υ* can always be chosen positive, up to a gauge transformation (see “Methods”). *γ*_*ij*_ = *x*, *y*, *z* denotes the bond type of bond 〈*ij*〉 (in a cubic environment each of the three types of honeycomb bonds is orthogonal to a different cubic axis *x*, *y*, *z* and may be thereby labeled, see Fig. 2), and *x* ± 1 = *y*, *z* etc. For example, if 〈*ij*〉 is a *z*-type bond,

2 $${H}_{\langle ij\rangle \in z}= \, 	{P}_{i}^{z}{P}_{j}^{z}\left[-\zeta +J\left({{\bf{S}}}_{i}\cdot {{\bf{S}}}_{j}+\beta {({{\bf{S}}}_{i}\cdot {{\bf{S}}}_{j})}^{2}\right)\right]\\ 	 -\upsilon \left[{T}_{i}^{y}{T}_{j}^{x}+{\rm{h.c.}}\right].$$

The physical relevance of the *ζ*, *J*, *β*, *υ* parameters is rooted in the relative geometry of the *t*_2*g*_ orbitals and honeycomb bonds. Indeed the, e.g., *d*_*xy*_ (or “*z*”-) orbitals at each end of a *z* bond have a large overlap, while all other overlaps are weak (see Fig. 2); the first term in Eq. (1) enforces precisely this concomitance of bond and orbital filling types across a bond. All terms in Eq. (1) arise from standard orbital-dependent superexchange mechanisms. They were derived in detail in refs. ^21,35^: the *υ* = 0 terms arise in the case of direct orbital overlap (180° bonds)^21^, while the *υ* term, which causes orbital fluctuations, is due to 90°-bond superexchange^35^. [Biquadratic interactions (*β* contribution to Eq. (1)) can be obtained at higher orders in *t*/*U*, where *t*, *U* are the usual Hubbard model parameters. They are nevertheless not crucial to the physics described in this manuscript, and mainly used as a crutch to tune the model to an exactly-soluble point]. Equation (1) with *υ* = 0 and *ζ* < 0 was studied in detail in ref. ^21^ and it was noted that loop states were not favored for the parameter values considered.

We now proceed to the analysis of this model.

### Orbital sector: fluctuating orbital loops

First, we set *J* = 0, and investigate the orbital part of the Hamiltonian, i.e.

3 $${H}_{{\rm{orb}}}=\mathop{\sum}\limits_{\langle ij\rangle }\left(-\zeta {P}_{i}^{{\gamma }_{ij}}{P}_{j}^{{\gamma }_{ij}}-\upsilon \left[{T}_{i}^{{\gamma }_{ij}-1}{T}_{j}^{{\gamma }_{ij}+1}+{\rm{h.c.}}\right]\right).$$

#### Static loops

**Fig. 3:**
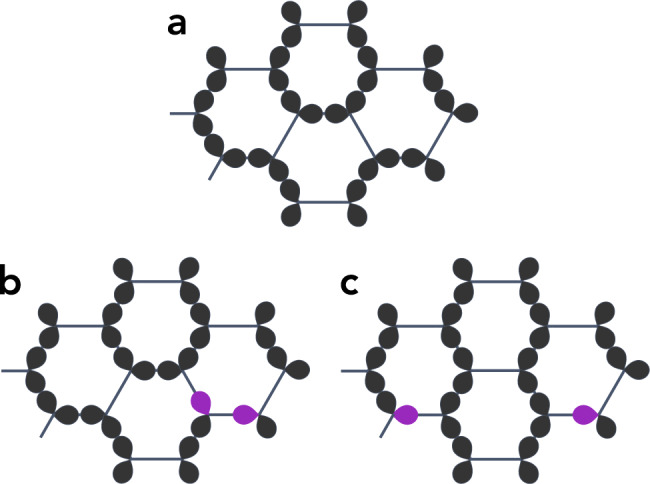
Pictorial representations of the loop and orbital configurations. **a** Orbital loop covering the lattice. **b**, **c** An elementary defect in the loop covering: **b** at the loop cut and **c** after part of the defect traveled. When the orbital loops are decorated by Haldane chains, the (purple) chain ends also carry a spin-1/2.

To begin, we also focus on *υ* = 0, in which case the Hamiltonian is exactly soluble. Indeed, *H*_orb_ then reduces to a (classical) Potts model ${H}_{0}=-\zeta {\sum }_{\langle ij\rangle }{P}_{i}^{{\gamma }_{ij}}{P}_{j}^{{\gamma }_{ij}}$^21^, with $[{P}_{i}^{\mu },{P}_{j}^{\nu }]=0$ and $[{H}_{0},{P}_{i}^{\mu }]=0$ ∀ *i*, *j*, *μ*, *ν*. For *ζ* > 0, because ${P}_{i}^{\gamma }$ measures the occupation of the *γ* orbital at site *i*, on each bond 〈*ij*〉 the energy is minimized when both orbitals *γ*_*ij*_ at each end are filled, in which case we say the bond is “covered”. Because there are two electrons per site, ${\sum }_{\gamma }{P}_{i}^{\gamma }=2$, the configuration where two covered bonds stem out of every site (forming a two-bond string) is favorable energetically (see Fig. 3a). The (fully-packed) loop coverings of the lattice implement this condition throughout the lattice and constitute the highly degenerate ground-state manifold of *H*_0_. [The loop covering manifold is isomorphic to the more familiar set of 1/3-plateau states, or 2/3-filling hard-core bosons, on the kagomé lattice by identifying a covered honeycomb *bond* with an “up” spin-1/2, or a hard-core boson, on the center of the bond.] Owing to the orthogonality of *t*_2*g*_ orbitals at the same site, all loop coverings are strictly orthogonal to one another^20^. This is in contrast to many dimer models where the dimers are two spin-1/2 singlets. The elementary excitations of *H*_0_ (which take one out of the loop covering manifold) are loop “cuts”: a loop is cut open, which creates nearby two (defect) bonds covered by one orbital rather than two or zero (see Fig. 3b, c). For *H*_0_, while a loop cut costs an energy *ζ* and locally creates two defect bonds, once created the two defect bonds may travel infinitely apart at no further energy cost. This is, of course, reminiscent of the classical spin ice problem, where a spin flip creates two monopoles that can (quasi-)freely separate.

#### Orbital fluctuations

We now consider a non-zero but small *υ* ≪ *ζ*. This gives dynamics to the loops, since now $[H,{P}_{i}^{\gamma }]\,\ne\, 0$. In degenerate perturbation theory in the *υ* = 0 manifold, the lowest-order effective Hamiltonian is

4 $${H}_{{\rm{eff}}}=-\frac{12{\upsilon }^{3}}{{\zeta }^{2}}\mathop{\sum}\limits_{\hexagon }{W}_{\hexagon},$$

where the sum is taken over all hexagons (or “plaquettes” of the lattice), and corresponds to the “flip” terms given pictorially by(microscopically, *t* = 12*υ*^3^/*ζ*^2^). In Eq. (5), in terms of the *T* operators

6 $${W}_{\hexagon }={\mathcal{P}}\ {T}_{1}^{x}{T}_{2}^{y}{T}_{3}^{z}{T}_{4}^{x}{T}_{5}^{y}{T}_{6}^{z}\ {\mathcal{P}}$$

where ${\mathcal{P}}$ is the projection onto the loop-covering manifold and where the sites 1, ..., 6 are defined around a hexagon as in Fig. 2a. Hexagons with alternating covered and empty bonds, such as those in Eq. (5), are called “flippable”.

In dimer problems, it is customary to introduce a term, the Rokhsar–Kivelson potential^36^, which counts the number of flippable plaquettes:which can be written *H*_RK_ = *V*∑_⎔_$\left[\mathop{\prod }\nolimits_{j = 0}^{2}{{\mathsf{P}}}_{1+2j}^{{\gamma }_{1+2j,2+2j}}{{\mathsf{P}}}_{2+2j}^{{\gamma }_{1+2j,2+2j}}+\right.\left.\mathop{\prod }\nolimits_{j = 0}^{2}{{\mathsf{P}}}_{2+2j}^{{\gamma }_{2+2j,3+2j}}{{\mathsf{P}}}_{3+2j}^{{\gamma }_{2+2j,3+2j}}\right]$, where ${{\mathsf{P}}}_{i}^{\mu }=1-{P}_{i}^{\mu }$. *H*_RK_ is primarily used as a “crutch” to gain insight from an accessible exactly-soluble point.

**Fig. 4:**
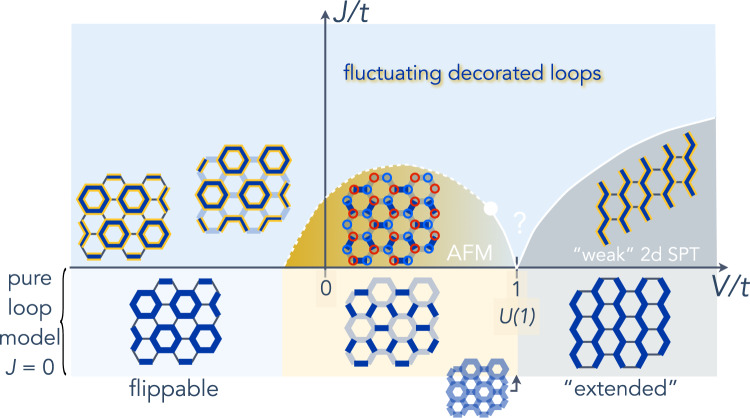
Phase diagram in 2*d* in the *V*/*t* − *J*/*t* plane (*t* = 12*υ*^3^/*ζ*^2^), for *J* ≥ 0 and *ζ* > 0. The phase diagram of the pure plain loop model is shown below the horizontal axis. In the small *J*/*t* region, the simplest situation of a single “intermediate” antiferromagnetic (AFM) phase is shown (a nematic valence bond solid-phase and multiple-phase transitions may also appear for some parameters, see “Methods” and Fig. 5). In the intermediate *J*/*t* region, the location and nature of the phase transitions are speculative. The solid and dashed white lines and the white dot represent putative second and first-order transitions, and critical end point, respectively. Thick *blue* lines represent the strength of orbital overlaps (darker blue means larger overlap), yellow contours Haldane chains and red and blue circles *S*^*z*^ = 1 and *S*^*z*^ = − 1 spins. As indicated in the text, SPT stands for symmetry-protected topological phase.

**Fig. 5:**
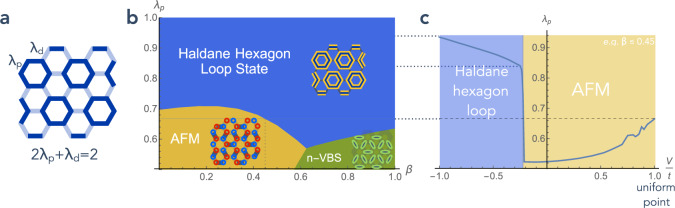
Variational phase diagram of the *S* = 1 spin model on the non-uniform honeycomb lattice. **a** The non-uniform exchange pattern considered, with ${\lambda }_{p},{\lambda }_{d}={\langle {P}_{i}^{{\gamma }_{ij}}{P}_{j}^{{\gamma }_{ij}}\rangle }_{p,d}$ bonds forming plaquette and dimer structures, respectively. Note that ${\sum }_{\gamma }{P}_{i}^{\gamma }=2$ and the loop constraint impose *λ*_*d*_ = 2(1 − *λ*_*p*_). **b** Phase diagram for the non-uniform bilinear-biquadratic model with the exchange pattern depicted in (**a**), in the *β* − *λ*_*p*_ plane, in a simple variational approach. AFM denotes the simple *S* = 1 antiferromagnet (red and blue circles represent *S*^*z*^ = 1 and *S*^*z*^ = −1 spin), n-VBS denotes a valence bond solid where the valence bonds have nematic symmetry, and the Haldane hexagon loop state is made of a crystal of hexagonal Haldane chains. **c** *λ*_*p*_ (shown in **a**) as a function of *V*/*t* as obtained from ref. ^38^. The labeling and coloring of the phases is given in the simplest case *β* ≲ 0.45.

The loop model in general, and $\tilde{H}={H}_{{\rm{flip}}}+{H}_{{\rm{RK}}}$ in particular, is in fact exactly dual to the dimer covering model obtained from the loop one by “swapping” the covered and empty bonds. The dimer model was studied in detail in several numerical works^37,38^, and the results adapted to our loop model are presented below the horizontal axis in Figs. 4 and  5a, c, which we now discuss. The phase diagram of $\tilde{H}$ contains an exactly soluble point, that where *V* = *t*, called the “RK point”, where the ground state is given by the equal-weight quantum superposition of all loop coverings of the lattice^36^. This state, where the loops fluctuate wildly, has an emergent *U*(1) (Coulombic) gauge field, and massive deconfined fractionalized excitations, whose classical analogs are the non-matching bonds obtained from loop cuts discussed above. It is a *U*(1) quantum orbital liquid, with a gapless (quadratic) photon mode.

**Fig. 6:**
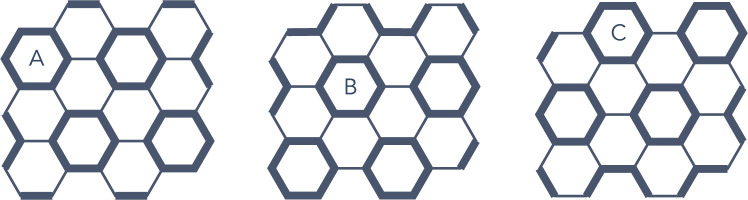
The three different hexagon loop crystals. The thicker bonds show the length-six hexagons loops, which form a triangular lattice.

It is a well-known result, however, that, in 2 + 1 dimensions, the deconfined phase of *U*(1) Coulombic gauge theories is unstable^39^, so that, in our model, the quantum orbital liquid regime does not exist as a phase in an extended region of the phase diagram, but survives only at the RK point. Away from the RK point, the system instead releases its entanglement, breaks symmetries and orders for both *V* > *t* and *V* < *t* into the phases shown below the horizontal axis in Fig. 4 and in Fig. 5a, c. At *V*/*t* > 1, the system immediately orders into ground states, which feature static “parallel” chains that extend through the whole system. For *V*/*t* < 1, the system first enters an “intermediate phase” where $0\, <\, \langle {P}_{i}^{{\gamma }_{ij}}{P}_{j}^{{\gamma }_{ij}}\rangle\, <\, 1$, before hitting a first-order phase transition below which the system favors one of the three “maximally flippable” “hexagon loop crystal” configurations (see “Methods” and Fig. 6).

We note that the model presented here can be generalized to three dimensions (on the hyperhoneycomb lattice^40^, which shares with the honeycomb lattice the same essential ingredients), where Coulombic phases of *U*(1) gauge theories are stable^39,41,42^. Details are beyond the scope of this paper, but will be addressed in an upcoming publication. Stable two-dimensional generalizations, such as those allowing for a ${{\Bbb{Z}}}_{2}$ spin liquid, are also possible.

### Spins

We now finally introduce the spins, i.e. consider *J* ≠ 0. First, we note that the spin operators appear only in

8 $${H}_{J}=\mathop{\sum}\limits_{\langle ij\rangle }{\tilde{J}}_{ij}({{\bf{S}}}_{i}\cdot {{\bf{S}}}_{j}+\beta {({{\bf{S}}}_{i}\cdot {{\bf{S}}}_{j})}^{2}),$$

where ${\tilde{J}}_{ij}=J{P}_{i}^{{\gamma }_{ij}}{P}_{j}^{{\gamma }_{ij}}$, which, when considered as a 1*d* problem with constant ${\tilde{J}}_{ij}\, > \, 0$ and −1 ≤ *β* ≤ 1, realizes the Haldane phase (and in particular the AKLT state described in the introduction at *β* = 1/3). Notably, the spin exchange is “modulated” by the operator ${P}_{i}^{{\gamma }_{ij}}{P}_{j}^{{\gamma }_{ij}}$, and vanishes when ${P}_{i}^{{\gamma }_{ij}}{P}_{j}^{{\gamma }_{ij}}=0$. Therefore, when the system forms orbital loops, the problem in spin space reduces to a collection of purely one-dimensional periodic *S* = 1 Hamiltonians, which are minimized by entering the Haldane phase. This leads to the appearance of new structures, namely Haldane-decorated loops, where each orbital loop subtends a Haldane chain, $|\hat{{\mathcal{L}}}\rangle =\left|{\mathcal{L}}\right\rangle \otimes \left|{\psi }_{{\rm{Haldane}}}\right\rangle$, where $\left|{\mathcal{L}}\right\rangle$ is an orbital loop, and $\left|{\psi }_{{\rm{Haldane}}}\right\rangle$ the Haldane spin ground state of the spins on the loop. Interestingly, the decoration in general introduces a length-dependent energy density. Indeed, away from *β* = 1/3, where the energy density (the energy per site, or bond) of periodic AKLT chains is independent of their length, the energy density of length-six loops is always smaller than that of longer loops (see Supplementary Fig. 2 for results obtained using density matrix renormalization group (DMRG)). In turn, this has consequences on the energetics of the loop coverings, which may become inequivalent.

#### Static coverings: *υ* = 0

In the absence of orbital fluctuations, i.e. when *υ* = 0, the ground state manifold of the pure orbital model is that of all loop coverings, as discussed above. In particular, all loop coverings are degenerate in energy, regardless of the distribution of their loop lengths. If we now consider *J* > 0 and *J* ≪ *ζ*, at first order in perturbation theory in *ζ*/*J* (*H*_*J*_ perturbs *H*_0_), spin states break the degeneracy of the loop coverings, following $\langle \hat{{\mathcal{C}}}| {H}_{J}| \hat{{\mathcal{C}}}\rangle$, where the $|\hat{{\mathcal{C}}}\rangle$ are the otherwise-degenerate decorated loop coverings. At *β* = 1/3, we retain an exact degeneracy between Haldane-decorated loop configurations, which all together form the ground state manifold, while, away from *β* = 1/3, $\langle \hat{{\mathcal{C}}}| {H}_{J}| \hat{{\mathcal{C}}}\rangle$ is only minimized when the system forms one of three equivalent “hexagon crystal” states where the lattice is covered by decorated loops of length six (see Fig. 5 for a variational phase diagram of the spin model on the non-uniform honeycomb lattice). This static-orbital regime corresponds to the infinite *J*/*t* limit on Fig. 4.

We now introduce the orbital kinetic terms, distinguishing between different *J*/*υ* regimes.

#### Large *J*/*υ* limit

In the large *J*/*t* limit, we first consider the Hamiltonian

9 $${H}_{{\rm{stat}}}=\mathop{\sum}\limits_{\langle ij\rangle }{P}_{i}^{{\gamma }_{ij}}{P}_{j}^{{\gamma }_{ij}}(-\zeta +J({{\bf{S}}}_{i}\cdot {{\bf{S}}}_{j}+\beta {({{\bf{S}}}_{i}\cdot {{\bf{S}}}_{j})}^{2})),$$

and introduce the kinetic terms *υ* in perturbation theory. Even with *J* ≠ 0 (and possibly *J* ~ *ζ*), the eigenstates of *H*_stat_ are still eigenstates of the ${P}_{i}^{\mu }={\hat{n}}_{i}^{\mu }$, and as discussed above ${\tilde{J}}_{ij}=J{P}_{i}^{{\gamma }_{ij}}{P}_{j}^{{\gamma }_{ij}}$ is only non-zero when ${\hat{n}}_{i}^{{\gamma }_{ij}}={\hat{n}}_{j}^{{\gamma }_{ij}}=1$. Therefore, ground states of *H*_stat_ belong to the set of decorated loop covering, so long as −*ζ* + *ϵ*_Hald cov_ < 0, where *ϵ*_Hald cov_ is the energy density of the collection of all pure Haldane chains in the covering. For example, for *β* = 0 (resp. *β* = 1/3), this is true for any *ζ* > *ϵ*_*L* = *∞*_(0) ≈ −1.40*J* (resp. *ζ* > *ϵ*_*L* = *∞*_(1/3) = −2/3*J*).

**Fig. 7:**
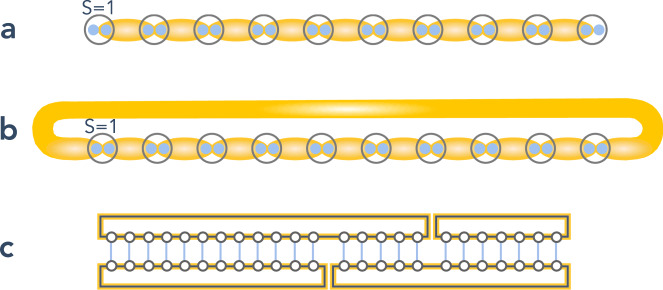
Pictorial representation of the AKLT chains and overlaps. **a** Open and **b** closed (periodic) AKLT chains, and **c** MPS representation of the transfer matrices for the overlap between different AKLT chains coverings.

When *β* = 1/3, the manifold of decorated loop coverings (which are ground states of *H*_stat_) is highly degenerate, and we call ${\mathfrak{P}}$ the projector onto this manifold. In perturbation theory in small *υ*/∣ − *ζ* + *ϵ*_Hald cov_∣, our analysis of the pure orbital model informs us that the lowest-order contribution arises at third order, provided $H^{\prime} ={\mathfrak{P}}({H}_{{\rm{flip}}}\otimes {{\mathbb{1}}}_{{\bf{S}}}){\mathfrak{P}}$ does not identically vanish. Indeed, *H*_flip_ is the lowest-order orbital-space Hamiltonian to take one orbital loop covering into another, and one must check that the corresponding Haldane loop coverings have non-zero overlap. Remarkably, we find that the overlap between two AKLT loop coverings (cov), which differ by a single plaquette flip is always equal to 1/4, up to exponentially small corrections in the lengths of the (rearranged) loops, i.e.

10 $$\langle {\hat{{\mathcal{C}}}}_{1}| ({H}_{{\rm{flip}}}\otimes {{\Bbb{1}}}_{{\bf{S}}})| {\hat{{\mathcal{C}}}}_{2}\rangle \propto \langle {{\rm{AKLTcov}}}_{1}| {{\rm{AKLTcov}}}_{2}\rangle \approx \frac{1}{4},$$

if ${{\mathcal{C}}}_{1}$ and ${{\mathcal{C}}}_{2}$ differ by a single plaquette flip, but regardless of how many loops are connected by this plaquette flip. [Also note that on a bipartite lattice, like the honeycomb, the sign of singlets across bonds can be unambiguously systematically chosen, in contrast to e.g. in ref. ^10^.] An exact result is obtained using the matrix product state (MPS) formalism (see “Methods”). In fact, Eq. (10) is a special case of $\langle \otimes {{\rm{AKLT}}}_{1}| \otimes {{\rm{AKLT}}}_{2}\rangle \approx 1/{2}^{{n}_{{\rm{cuts}}}-1}$, where *n*_cuts_ is the number of loop cuts needed to connect $|\otimes {{\rm{AKLT}}}_{1,2}\rangle$ (see Fig. 7c). This momentous result is a consequence of the ultra-short-range entanglement of the AKLT state, which is “close” to being a product of single-site states.

Away from *β* = 1/3, but within the Haldane phase (∣*β*∣ ≤ 1), we expect the same results to hold since entanglement properties are characteristic of a phase. Therefore, at large *J*/*t*, and for *β* = 1/3 and *V*/*t* = 1/4 (decorated RK point), the system is “close to” [The overlap Eq. (10) is only approximately equal to 1/4, and depends—albeit exponentially—on loop length; this could, in fact, bear additional interesting consequences^43^.] a *U*(1) phase with spinful fractional excitations, in the sense that it contains large fluctuating Haldane-decorated loops. This is a state our model Eq. (1) was designed to achieve. The lowest-energy excitations are either loop cuts (necessarily accompanied by a Haldane chain cut) or pure Haldane chain excitations, depending on the distribution of loop length and values of *ζ* and *β* (see Supplementary Table I). Local loop cuts generate two orbital chain ends, which are decorated by spin-1/2. In three dimensions, where the orbital *U*(1) deconfined phase survives away from the RK point, these chain ends are deconfined spinons.

#### Small *J*/*υ* limit

In the small *J*/*t* limit, the orbital-only model, i.e. *H*_orb_ from Eq. (8), is solved first and spin exchange (*J*) is then introduced perturbatively. This results in an effective exchange pattern for the spins. More precisely, when *J* = 0, spin space is completely degenerate, but the orbital ground state is a priori unique (or discretely degenerate due to symmetry-related states). Therefore, upon introducing *J*, in degenerate perturbation theory, we have, at first order,

11 $${{H}_{\mathrm{{eff}}}^{\prime \prime}}	={\mathop{\sum}\limits_{\langle {ij}\rangle }}{\mathfrak{p}}[J{P}_{i}^{{\gamma }_{ij}}{P}_{j}^{{\gamma }_{ij}}({{\bf{S}}}_{i}\cdot {{\bf{S}}}_{j}+\beta {({{\bf{S}}}_{i}\cdot {{\bf{S}}}_{j})}^{2})]{\mathfrak{p}}\\ 	={\mathop{\sum}\limits_{\langle {ij}\rangle }}\langle {P}_{i}^{{\gamma }_{ij}}{P}_{j}^{{\gamma }_{ij}}\rangle J({{\bf{S}}}_{i}\cdot {{\bf{S}}}_{j}+\beta {({{\bf{S}}}_{i}\cdot {{\bf{S}}}_{j})}^{2}),$$

where ${\mathfrak{p}}$ is the projector onto the *J* = 0 ground state, and $\left\langle\, .\right\rangle$ is the expectation value taken in this ground state. We obtain the phase diagram Fig. 4 in the small *J*/*υ* limit within the pure loop model by (i) solving the non-uniform bilinear-biquadratic model Eq. (11) within a simple variational approach (see Fig. 5b) and “Methods” text for a detailed derivation and (ii) using the results from ref. ^38^. Notably, the Haldane loop decoration in the extended phase at large *V*/*t* > 0 gives way to a two-dimensional weak SPT phase, with neutral Kramers doublet (time-reversal symmetry is also preserved in that phase) edge states (the edge orbitals decorated by spin-1/2 degrees of freedom) protected by translational symmetry, provided the boundaries are appropriately chosen (and, strictly speaking, provided a weak coupling at the boundary exists). It is noteworthy that this phase is realized spontaneously, i.e. this is not an explicit chain-stacking construction. To our knowledge, this is the first such example in the literature.

In this regime (*ζ* > 0, *ζ* ≫ *J* > *υ*) low-energy excitations are expected to occur in the spin sector. In the antiferromagnetic (AFM) phase, those are simply the conventional spin flips. In the decorated chain phases, the elementary excitations are those of the (gapped) Haldane chains. Remarkably, in the length-six loop state, which is a product state of decorated hexagon loops, the excitations are local, but a weak coupling between the hexagons (e.g. when *υ* ≠ 0) will lead to slightly dispersive “Haldane-gap waves” observable, e.g. in neutron scattering.

The results derived above are summarized in the phase diagram in Fig. 4.

## DISCUSSION

In summary, we have exposed a physical mechanism for the realization of fluctuating Haldane chains in spin-orbital models in two dimensions. To do so, we presented a realistic and analytically tractable spin-orbital Hamiltonian on the honeycomb lattice, with a rich phase diagram, featuring exotic phases built out of Haldane chains. Among those is a translational SPT phase, with spin-1/2 edge excitations, a Haldane hexagon loop “crystal”, with “Haldane-gap wave” excitations, and a regime with fluctuating Haldane chains coupled to underlying “orbital loops”. On the three-dimensional hyperhoneycomb lattice, the latter becomes a Coulombic quantum spin-orbital liquid, a unique example in the spin-orbital literature of a controllable model where both the spin and orbital sectors are “disordered”. Moreover, supplementing the model with additional terms is likely to allow accessing more phases and possibly interesting phase transitions. In fact, many more avenues—in several different fields—will be worth exploring further. For example, the QSL can be induced not only by taking the model to three dimensions but also by turning it into a ${{\Bbb{Z}}}_{2}$ liquid. The variation of the parameter *ζ* or the number of electrons per site may also lead to interesting problems and phase transitions. In general, the highlighted mechanism will hopefully be an important stepping stone for future studies to realize Haldane chains and other low-dimensional structures in higher dimensions.

Most exciting would certainly be the discovery in real materials of some of the phenomena described here. The model presented here is relevant to insulating honeycomb materials with two electrons in degenerate *t*_2*g*_ orbitals and large Hund’s coupling to enforce *S* = 1. In practice, materials need to have (at least approximate) cubic symmetry, weak spin–orbit coupling and large direct orbital overlap. Therefore, materials based on Ru, Ni, V, etc. naively appear as potential candidates. Regardless, it will be important and interesting to study the breaking of any of these constraints, through, e.g. spin–orbit coupling or symmetry lowering, inevitable at some level in real materials. Magneto-elastic coupling should also be investigated. It might well play a role similar to the *V* term in stabilizing the “extended” or “flippable” phases.

## Methods

### Effective orbital operators

#### Construction of the states and operators

Let us consider two electrons per site, and degenerate *t*_2*g*_ (*d*_*xy*_, *d*_*xz*_ and *d*_*yz*_) orbitals at each site, and a very large intra-orbital *U*, so that there is only one electron per orbital, a large Hund’s coupling *J*_H_ so that *S* = 1, and no spin–orbit coupling. We define the states of the three-dimensional orbital space to be $\left|x\right\rangle$, $|y\rangle$ and $\left|z\right\rangle$, such that, if $\left|0\right\rangle$ is the Fock space vacuum at a given site for spinless electrons and ${c}_{\mu \nu }^{\dagger }$ creates a spinless electron in orbital *d*_*μν*_:

12 $$\left|\gamma \right\rangle ={c}_{\gamma +1,\gamma +2}({c}_{yz}^{\dagger }{c}_{xz}^{\dagger }{c}_{xy}^{\dagger }\left|0\right\rangle ).$$

The normalization is chosen such that $\langle \gamma | \gamma ^{\prime} \rangle ={\delta }_{\gamma ,\gamma ^{\prime} }$. Note that an unimportant (convention-dependent) choice of phase was made.

We now define the operators *L*^*c*^, *c* = *x*, *y*, *z* according to

13 $${L}^{c}=\frac{-i}{2}\mathop{\sum}\limits_{a,b}{\epsilon }_{abc}(\left|a\right\rangle \left\langle b| -| b\right\rangle \left\langle a\right|),$$

which may be rewritten as:

14 $${L}^{\gamma }=-i\left[\left|\gamma +1\right\rangle \left\langle \gamma -1| -| \gamma -1\right\rangle \left\langle \gamma +1\right|\right].$$

These operators obey ${L}^{\gamma }\left|\gamma \right\rangle =0$ and **L**^2^ = 2. One may check that these operators obey the angular momentum algebra commutation relations. To form a complete basis of Hermitian operators acting in our three-dimensional space, we need six more Hermitian operators, which we choose to be ${({L}^{a})}^{2}={P}^{a}$ and {*L*^*a*^, *L*^*b*^} = *T*^*c*^, *a* ≠ *b*. Note that

15 $${P}^{\gamma }	={({L}^{\gamma })}^{2}\\ 	=\left|\gamma +1\right\rangle \left\langle \gamma +1| +| \gamma -1\right\rangle \left\langle \gamma -1| =1-| \gamma \right\rangle \left\langle \gamma \right|$$

and

16 $${T}^{\gamma }=\{{L}^{\gamma +1},{L}^{\gamma -1}\}=-\left(\left|\gamma +1\right\rangle \left\langle \gamma -1| +| \gamma -1\right\rangle \left\langle \gamma +1\right|\right).$$

#### Effective operators as rotation and projection operators

${L}^{\gamma }\left|\gamma \right\rangle =0$ so the projection operator onto the $\left|\gamma \right\rangle$ component is ${{\mathsf{P}}}_{\gamma }=1-{({L}^{\gamma })}^{2}$. The projection operator in Eq. (1) of the main text is ${P}_{\gamma }=1-{{\mathsf{P}}}_{\gamma }={({L}^{\gamma })}^{2}$. In particular,

17 $${P}_{\gamma }\left|\gamma \right\rangle =0,\qquad {P}_{\gamma }\left|\gamma \pm 1\right\rangle =\left|\gamma \pm 1\right\rangle .$$

Disregarding phase factors:

18 $$\left\{\begin{array}{l}{L}^{\gamma }\left|\gamma \right\rangle =0\\ {T}^{\gamma }\left|\gamma \right\rangle =0\end{array}\right.,\qquad \left\{\begin{array}{l}{L}^{\gamma }\left|\gamma \pm 1\right\rangle \propto \left|\gamma \mp 1\right\rangle \\ {T}^{\gamma }\left|\gamma \pm 1\right\rangle \propto \left|\gamma \mp 1\right\rangle \end{array}\right..$$

### Haldane covering overlaps at the AKLT point: MPS formalism

The AKLT loop covering overlaps are calculated with the MPS formalism. An exact representation of the AKLT wavefunction is given as an MPS:

19 $${\left|\psi \right\rangle }_{{\rm{AKLT}}}=\mathop{\sum}\limits_{\{{\sigma }_{i}=0,\pm 1\}}{\rm{Tr}}\left[{\mathsf{M}}({\sigma }_{1})\cdots {\mathsf{M}}({\sigma }_{N})\right]\left|{\sigma }_{1}\cdots {\sigma }_{N}\right\rangle ,$$

when the chain is a closed loop of length *N* (we use the notations from ref. ^44^) with the following matrices M:

20 $$\left\{\begin{array}{lll}{\mathsf{M}}(\sigma =0)&=&-\sqrt{\frac{1}{3}}{\sigma }^{z}\\ {\mathsf{M}}(\sigma =\pm\! 1)&=&-\sqrt{\frac{2}{3}}{\sigma }^{\pm }\end{array}\right.,$$

with *σ*^*μ*^ the Pauli matrices, and with the norm ∣*ψ*∣^2^:

21 $$\langle \psi | \psi \rangle ={\rm{Tr}}{{\mathsf{T}}}^{N},\qquad \,{\text{where}}\,\qquad {\mathsf{T}}=\mathop{\sum}\limits_{\sigma =\pm 1,0}{{\mathsf{M}}}^{* }(\sigma )\otimes {\mathsf{M}}(\sigma ).$$

We then compute the overlap between different types of coverings connected by a single plaquette flip. The difference between those configurations lies purely in the number and lengths of the loops “touching” the flippable plaquette of interest, before and after the plaquette flip. Within the MPS formalism, these overlaps are given by:

**Case 1** Three loops connected to one:

22 $$	\langle {{\rm{AKLT}}}_{0}| {{\rm{AKLT}}}_{1},{{\rm{AKLT}}}_{2},{{\rm{AKLT}}}_{3}\rangle \\ \,	\qquad =\frac{{\rm{Tr}}[{{\mathsf{T}}}_{1}^{{n}_{1}}{{\mathsf{T}}}_{2}^{{n}_{2}}{{\mathsf{T}}}_{3}^{{n}_{3}}]}{\sqrt{{\rm{Tr}}[{{\mathsf{T}}}^{{n}_{0}}]{\rm{Tr}}[{\tilde{{\mathsf{T}}}}_{1}^{{n}_{1}}{\tilde{{\mathsf{T}}}}_{2}^{{n}_{2}}{\tilde{{\mathsf{T}}}}_{3}^{{n}_{3}}]}}\\ \,	\qquad =\frac{(3+{3}^{{n}_{3}})(13+2\sqrt{2}\ {3}^{{n}_{1}}+{3}^{{n}_{2}}(3-\sqrt{2}+2\cdot {3}^{{n}_{1}}))}{4\sqrt{2}\sqrt{(5+{3}^{{n}_{2}}(1+2\cdot {3}^{{n}_{1}}))(3+{3}^{{n}_{3}})(3+{3}^{{n}_{0}})}}$$

using the configuration from “Methods” and Fig. 8a, where *n*_0_ = *n*_1_ + *n*_2_ + *n*_3_,

23 $${{\mathsf{T}}}_{1}	=\mathop{\sum}\limits_{\sigma =0,\pm 1}{{\mathsf{M}}}^{* }(\sigma )\otimes {\mathsf{M}}(\sigma )\otimes {\bf{1}}\otimes {\bf{1}}\\ {{\mathsf{T}}}_{2}	=\mathop{\sum}\limits_{\sigma =0,\pm 1}{{\mathsf{M}}}^{* }(\sigma )\otimes {\bf{1}}\otimes {\mathsf{M}}(\sigma )\otimes {\bf{1}}\\ {{\mathsf{T}}}_{3}	=\mathop{\sum}\limits_{\sigma =0,\pm 1}{{\mathsf{M}}}^{* }(\sigma )\otimes {\bf{1}}\otimes {\bf{1}}\otimes {\mathsf{M}}(\sigma )$$

and

24 $${\tilde{{\mathsf{T}}}}_{1}	=\mathop{\sum}\limits_{\sigma =0,\pm 1}{{\mathsf{M}}}^{* }(\sigma )\otimes {\bf{1}}\otimes {\bf{1}}\otimes {\mathsf{M}}(\sigma )\otimes {\bf{1}}\otimes {\bf{1}}\\ {\tilde{{\mathsf{T}}}}_{2}	=\mathop{\sum}\limits_{\sigma =0,\pm 1}{\bf{1}}\otimes {{\mathsf{M}}}^{* }(\sigma )\otimes {\bf{1}}\otimes {\bf{1}}\otimes {\mathsf{M}}(\sigma )\otimes {\bf{1}}\\ {\tilde{{\mathsf{T}}}}_{3}	=\mathop{\sum}\limits_{\sigma =0,\pm 1}{\bf{1}}\otimes {\bf{1}}\otimes {{\mathsf{M}}}^{* }(\sigma )\otimes {\bf{1}}\otimes {\bf{1}}\otimes {\mathsf{M}}(\sigma )$$

**Case 2** Two loops connected to another two loops:

25 $$	\left\langle {{\mathrm{AKLT}}_1,{\mathrm{AKLT}}_2\left| {{\mathrm{AKLT}}_3} \right.,{\mathrm{ALKT}}_4} \right\rangle \\ 	\qquad= \frac{{{\mathrm{Tr}}[ {{\mathrm{T}}_1^{\prime ^{n_3^\prime }}{\mathrm{T}}_2^{\prime ^{n_1^\prime - n_3^\prime }}{\mathrm{T}}_3^{\prime ^{n_2^\prime }}} ]}}{{\sqrt {{\mathrm{Tr}}[ {\tilde {\mathrm{T}}_1^{\prime n_1^\prime }\tilde {\mathrm{T}}_2^{\prime n_2^\prime }} x]{\mathrm{Tr}}[ {\tilde {\mathrm{T}}_1^{\prime n_3^\prime }\tilde {\mathrm{T}}_2^{\prime n_4^\prime }}]} }}\\ 	\qquad= \frac{{\left( {3 + 3^{n_2^\prime }} \right)\left( {13 + 2\sqrt 2 \,3^{n_3^\prime } + 3^{n_1^\prime - n_3^\prime }\left( {3 - \sqrt 2 + 2 \cdot 3^{n_3^\prime }} \right)} \right)}}{{8\sqrt {\left( {3 + 3^{n_3^\prime }} \right)\left( {3 + 3^{n_2^\prime }} \right)\left( {3 + 3^{n_1^\prime }} \right)\left( {3 + 3^{n_1^\prime + n_2^\prime - n_3^\prime }} \right)} }}$$

using the configuration from “Methods” and Fig. 8b, with ${n}_{1}^{\prime}+{n}_{2}^{\prime}={n}_{3}^{\prime}+{n}_{4}^{\prime}$, and where

26 $${{\mathsf{T}}}_{1}^{\prime}	=\mathop{\sum}\limits_{\sigma =0,\pm 1}{{\mathsf{M}}}^{* }(\sigma )\otimes {\mathsf{M}}(\sigma )\otimes {\bf{1}}\otimes {\bf{1}}\\ {{\mathsf{T}}}_{2}^{\prime}	=\mathop{\sum}\limits_{\sigma =0,\pm 1}{{\mathsf{M}}}^{* }(\sigma )\otimes {\bf{1}}\otimes {\mathsf{M}}(\sigma )\otimes {\bf{1}}\\ {{\mathsf{T}}}_{3}^{\prime}	=\mathop{\sum}\limits_{\sigma =0,\pm 1}{\bf{1}}\otimes {\bf{1}}\otimes {\mathsf{M}}(\sigma )\otimes {{\mathsf{M}}}^{* }(\sigma )$$

and

27 $${\tilde{{\mathsf{T}}}}_{1}^{\prime}	=\mathop{\sum}\limits_{\sigma =0,\pm 1}{{\mathsf{M}}}^{* }(\sigma )\otimes {\bf{1}}\otimes {\mathsf{M}}(\sigma )\otimes {\bf{1}}\\ {\tilde{{\mathsf{T}}}}_{2}^{\prime}	=\mathop{\sum}\limits_{\sigma =0,\pm 1}{\bf{1}}\otimes {{\mathsf{M}}}^{* }(\sigma )\otimes {\bf{1}}\otimes {\mathsf{M}}(\sigma ).$$

The overlaps in “Methods” and Eqs. (22) and (25) take the form of a dominant 1/4 contribution and exponentially decaying terms. This (dominating) length-independent contribution is simply equal to the overlap of “neighboring” *S* = 1/2 spins from different chains (see “Methods” and Fig. 8c), without the *S* = 1 on-site projections, and may be empirically understood from the very short-range entanglement in the AKLT wavefunction. In fact, 1/4 is a special case of a more general formula according to which the overlap of two coverings where *n*_cuts_ loop cuts are needed to reconnect them is equal to $1/{2}^{{n}_{{\rm{cuts}}}-1}$. For example, if $\left|{\psi }_{1}\right\rangle$ and $\left|{\psi }_{2}\right\rangle$ are as depicted in “Methods” and Fig. 8c, then

28 $$\left|{\psi }_{1}\right\rangle 	= \, \frac{1}{2}\left(\left|{\uparrow }_{1}{\downarrow }_{2}\right\rangle -\left|{\downarrow }_{1}{\uparrow }_{2}\right\rangle \right)\left(\left|{\uparrow }_{3}{\downarrow }_{4}\right\rangle -\left| {\downarrow }_{3}{\uparrow }_{4}\right\rangle \right)\\ 	= \, \frac{1}{2}\left[\left|{\uparrow }_{1}{\downarrow }_{2}{\uparrow }_{3}{\downarrow }_{4}\right\rangle +\left| {\downarrow }_{1}{\uparrow }_{2}{\downarrow }_{3}{\uparrow }_{4}\right\rangle -\left|{\uparrow }_{1}{\downarrow }_{2}{\downarrow }_{3}{\uparrow }_{4}\right\rangle -\left|{\downarrow }_{1}{\uparrow }_{2}{\uparrow }_{3}{\downarrow }_{4}\right\rangle \right]$$

and

29 $$\left\langle {\psi }_{2}\right|	= \, \frac{1}{2}\left(\left\langle {\uparrow }_{1}{\downarrow }_{4} \right|-\left\langle {\downarrow }_{1}{\uparrow }_{4} \right|\right)\left(\left\langle {\uparrow }_{3}{\downarrow }_{2} \right|-\left\langle {\downarrow }_{3}{\uparrow }_{2} \right|\right)\\ 	= \, \frac{1}{2}\left[\left\langle {\uparrow }_{1}{\downarrow }_{2}{\uparrow }_{3}{\downarrow }_{4} \right|+\left\langle {\downarrow }_{1}{\uparrow }_{2}{\downarrow }_{3}{\uparrow }_{4} \right|-\left\langle {\uparrow }_{1}{\uparrow }_{2}{\downarrow }_{3}{\downarrow }_{4} \right|-\left\langle {\downarrow }_{1}{\downarrow }_{2}{\uparrow }_{3}{\uparrow }_{4} \right|\right]$$

and so

30 $$\langle {\psi }_{2}| {\psi }_{1}\rangle =\frac{1}{2},$$

consistent with the fact that two loop cuts are needed to go from the upper to the lower MPS, and vice versa.

**Fig. 8:**
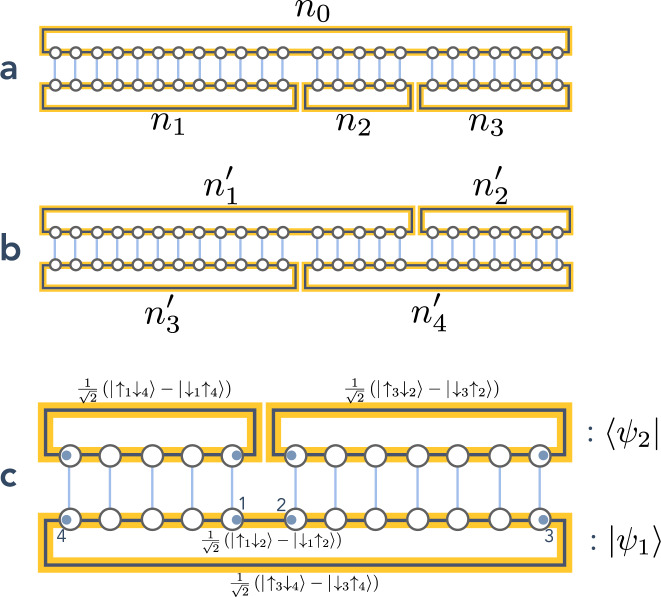
Transfer matrix overlap representation in the matrix product state formalism. **a** Overlap between three loops and one loop, **b** overlap between two sets of two loops and **c** overlap between one loop and two loops, emphasizing the role of the fractional degrees of freedom near the cuts.

### Details of the spin model phase diagram on the non-uniform honeycomb lattice

Here, we take a simple variational approach and calculate the energy of three kinds of states, for varying *β* and *λ*_*p*_ for the pattern depicted in “Methods” and Fig. 5a. The three types of states are the simple antiferromagnet, a valence bond solid where identical singlets or “nematic valence bonds” (see below) lie on *λ*_*d*_ bonds, and a Haldane hexagon crystal where length-six Haldane chains form along *λ*_*p*_ bonds. We consider the Hamiltonian

31 $$H	={\lambda }_{p}J\mathop{\sum}\limits_{\langle ij\rangle \in p}({{\bf{S}}}_{i}\cdot {{\bf{S}}}_{j}+\beta {({{\bf{S}}}_{i}\cdot {{\bf{S}}}_{j})}^{2})\\ 	\;\; +{\lambda }_{d}J\mathop{\sum}\limits_{\langle ij\rangle \in d}({{\bf{S}}}_{i}\cdot {{\bf{S}}}_{j}+\beta {({{\bf{S}}}_{i}\cdot {{\bf{S}}}_{j})}^{2}).$$

The approach could be readily refined to the next simplest level of variational approach by considering more general MPS network states (e.g. non-uniform along a plaquette, still with bond dimension 2 to keep it simple), but we deem it unnecessary for our purpose, as we see below.

The energy per bond in the simple antiferromagnet, where the state of a bond is of the form $\left|{\rm{AFM}}\right\rangle =\left|1-1\right\rangle$, is given by

32 $${\epsilon }^{{\rm{AFM}}}({\lambda }_{p},\beta )=\frac{J}{3}(2{\lambda }_{p}+{\lambda }_{d})(-1+2\beta )=\frac{2J}{3}(2\beta -1).$$

The energies of length-six Haldane chains at various *β*’s obtained with exact diagonalization are given in Supplementary Table I. To obtain the energy of the *λ*_*d*_ bonds, we use the eigenvectors, and find that it is simply proportional to *β*,

33 $${\epsilon }^{{\rm{Haldane}}}({\lambda }_{p},\beta )	=\frac{J}{3}\left(2{\lambda }_{p}{\epsilon }_{6}(\beta )+\beta {\lambda }_{d}\frac{4}{3}\right)\\ 	=\frac{2J}{3}\left(\frac{4\beta }{3}+{\lambda }_{p}\left({\epsilon }_{6}(\beta )-\frac{4\beta }{3}\right)\right).$$

The last phases we consider are the product states of (identical) *λ*_*d*_-bond states, which would minimize the energy of the Hamiltonian on a single *isolated* bond. For *β* ≤ 1/3, these *λ*_*d*_ bond states are *S* = 0 states, $\left|{\rm{VB}}\right\rangle =\frac{1}{\sqrt{3}}(\left|1-1\right\rangle -\left|00\right\rangle +\left|-11\right\rangle )$, in which case the energy density of their product states on the lattice is

34 $${\epsilon}^{{\rm{VB}}}({\lambda}_{p},\beta )	=\frac{J}{3}\left(2{\lambda }_{d}\left(2\beta -1\right)+\frac{3\beta }{8}{\lambda }_{p}\right)\\ 	=\frac{4J}{9}\left(-3+3{\lambda }_{p}+6\beta -4\beta {\lambda }_{p}\right).$$

For *β* ≥ 1/3, the ground states are the three *S* = 1 states, whose degeneracy is partially lifted by considering the *λ*_*p*_ couplings. The minimal accessible energy density is realized in the product states where the valence bonds are “nematic”, i.e. do not break time-reversal symmetry, but break rotation symmetry, such as $\left|{\rm{n}}\mbox{-}{{\rm{VB}}}_{0}\right\rangle =\frac{1}{\sqrt{2}}(\left|1-1\right\rangle +\left|-11\right\rangle )$ (which is the *S* = 1, *S*^*z*^ = 0 two-spin state, sometimes called $\left|z\right\rangle$ in the spin nematic literature), and the energy density is

35 $${\epsilon }^{{\rm{n}}\mbox{-}{\rm{VB}}}({\lambda }_{p},\beta )	=\frac{J}{3}\left({\lambda }_{d}(\beta -1)+3\beta {\lambda }_{p}\right)\\ 	=\frac{J}{3}(-2+2{\lambda }_{p}+2\beta +\beta {\lambda }_{p}).$$

When *λ*_*p*_ ≠ 0, which is always the case in our problem, by equating *ϵ*^VB^ and *ϵ*^n-VB^, we see immediately that the boundary between $\left|{\rm{VB}}\right\rangle$ and the $\left|{\rm{n}}\mbox{-}{\rm{VB}}\right\rangle$ states shifts away from *β* = 1/3.

Finally, within this simple variational approach, and for 1/2 ≤ *λ*_*p*_ ≤ 1, we find that the system is never in the singlet VBS phase, but that the system is in the AFM phase if

36 $$\left\{\begin{array}{l}{\lambda }_{p}\ge \frac{4\beta -1}{2+\beta }\\ {\lambda }_{p}\le \frac{3-2\beta }{4\beta -3{\epsilon }_{6}(\beta )}\end{array}\right.,$$

the system is in the n-VBS phase if

37 $$\left\{\begin{array}{l}{\lambda }_{p}\le \frac{4\beta -1}{2+\beta }\\ {\lambda }_{p}\le \frac{2(1+\beta )}{2+5\beta -3{\epsilon }_{6}(\beta )}\end{array}\right.,$$

and the system is in the Haldane crystal phase if

38 $$\left\{\begin{array}{l}{\lambda }_{p}\ge \frac{3-2\beta }{4\beta -3{\epsilon }_{6}(\beta )}\\ {\lambda }_{p}\ge \frac{2(1+\beta )}{2+5\beta -3{\epsilon }_{6}(\beta )}\end{array}\right..$$

Using these relations, we draw the approximate phase diagram shown in “Methods” and Fig. 5b. The results are consistent with those obtained for the uniform exchange bilinear–biquadratic model in refs. ^45,46^.

## Supplementary information

Supplementary Information

Peer Review File

## Data Availability

Source data are provided with this paper.
