# Supplementary material for: Quantum loop states in spin-orbital models on the honeycomb lattice
Source: Nat Commun. 2021 May 21;12:3004. doi: 10.1038/s41467-021-23033-y (PMC8139991; doi:10.1038/s41467-021-23033-y)
Supplement: Supplementary file 1 — Supplementary Information [file 41467_2021_23033_MOESM1_ESM.pdf]

Quantum loop states in spin-orbital models on the honeycomb lattice

Lucile Savary[1, 2]
[1] Department of Physics, Massachusetts Institute of Technology, 77 Massachusetts Ave., Cambridge, MA 02139
[2] Université de Lyon, École Normale Supérieure de Lyon, Université Claude Bernard Lyon I, CNRS, Laboratoire de physique, 46, allée d'Italie, 69007 Lyon*
(Dated: April 5, 2021)

SUPPLEMENTARY INFORMATION

Supplementary Note 1. Spin-orbital model

In the orbital sector, the coupling Hamiltonian between two sites 1 and 2 connected by an x-type bond, as defined in the Methods, takes the form:

$$H_{12}^{orb} = \begin{pmatrix} L_1^{x2} & L_1^{y2} & L_1^{z2} \end{pmatrix} \begin{pmatrix} \mathcal{J}_1 & \mathcal{J}_4 & \mathcal{J}_4 \\ \mathcal{J}_4 & \mathcal{J}_2 & \mathcal{J}_3 \\ \mathcal{J}_4 & \mathcal{J}_3 & \mathcal{J}_2 \end{pmatrix} \begin{pmatrix} L_2^{x2} \\ L_2^{y2} \\ L_2^{z2} \end{pmatrix} + \begin{pmatrix} L_1^x & L_1^y & L_1^z \end{pmatrix} \begin{pmatrix} \mathcal{J}_5 & \mathcal{J}_8 & -\mathcal{J}_8 \\ \mathcal{J}_8 & \mathcal{J}_6 & \mathcal{J}_7 \\ -\mathcal{J}_8 & \mathcal{J}_7 & \mathcal{J}_6 \end{pmatrix} \begin{pmatrix} L_2^x \\ L_2^y \\ L_2^z \end{pmatrix}$$
$$+ \begin{pmatrix} \{L_1^y, L_1^z\} & \{L_1^x, L_1^z\} & \{L_1^x, L_1^y\} \end{pmatrix} \begin{pmatrix} \mathcal{J}_9 & \mathcal{J}_{12} & -\mathcal{J}_{12} \\ \mathcal{J}_{12} & \mathcal{J}_{10} & \mathcal{J}_{11} \\ -\mathcal{J}_{12} & \mathcal{J}_{11} & \mathcal{J}_{10} \end{pmatrix} \begin{pmatrix} \{L_2^y, L_2^z\} \\ \{L_2^x, L_2^z\} \\ \{L_2^x, L_2^y\} \end{pmatrix}. \quad (1)$$

In Eq. (1) of the main text, if $J = 0$, $\mathcal{J}_1 = -\zeta$, $\mathcal{J}_7 = -\upsilon_1$, $\mathcal{J}_{11} = -\upsilon_2$, and all others zero. While the number of parameters is large (12), many of them are expected to be zero, physically. For example, it is unclear whether it is possible to obtain terms which involve a single power of angular momentum at each site from standard superexchange calculations [1, 2].

Upon introducing the spin degrees of freedom, in principle, each independent coefficient may be a spin Hamiltonian of the form (for no spin-orbit coupling):

$$\mathcal{J}_p = A_p + B_p \mathbf{S}_1 \cdot \mathbf{S}_2 + C_p (\mathbf{S}_1 \cdot \mathbf{S}_2)^2, \quad (2)$$

where $p = 1, .., 12$ labels the independent terms. In Eq. (1) of the main text, we took $\mathcal{J}_1 = -\zeta + J(\mathbf{S}_1 \cdot \mathbf{S}_2 + \beta(\mathbf{S}_1 \cdot \mathbf{S}_2)^2)$, i.e. $A_1 = -\zeta$, $B_1 = J$, $C_1 = J\beta$, and $\mathcal{J}_7 = -\upsilon_1$, i.e. $A_7 = -\upsilon_1$ and $B_7 = C_7 = 0$, and $\mathcal{J}_{11} = -\upsilon_2$, i.e. $A_{11} = -\upsilon_2$ and $B_{11} = C_{11} = 0$ and all other terms zero.

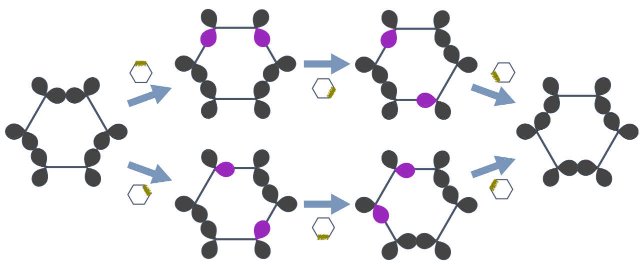

Supplementary Fig. 1. **Flippable plaquette adventure through third-order perturbation theory.** The small hexagons next to the arrows show the bond on which $H_{\rm kin}$ is applied at that order. The orbitals highlighted in purple do not have a matching orbital on the bond on which they lie.

Supplementary Note 2. Details of perturbation theory

Here we focus on orbital space, i.e. set $J = 0$, and give a few details for the degenerate perturbation theory in

$$H_{\rm kin} = -\upsilon \sum_{\langle ij \rangle} (T_i^{\gamma_{ij}-1} T_j^{\gamma_{ij}+1} + T_i^{\gamma_{ij}+1} T_j^{\gamma_{ij}-1}) \quad (3)$$

* lucile.savary@ens-lyon.fr

onto the manifold of loop coverings of the lattice, valid when $\zeta > 0$ and $\zeta \gg \upsilon$. The effective Hamiltonian is

$$H_{\rm eff} = \mathcal{P} H_{\rm kin} \frac{1-\mathcal{P}}{H-E_0} H_{\rm kin} \frac{1-\mathcal{P}}{H-E_0} \cdots \frac{1-\mathcal{P}}{H-E_0} H_{\rm kin} \mathcal{P}, \qquad (4)$$

where $H_{\rm kin}$ appears as many times as the order in perturbation theory.

Consider a "flippable" plaquette. Acting once with $H_{\rm kin}$ on any bond which belongs to the plaquette creates two "defect" bonds (this configuration does not belong to the loop covering manifold), with the new plaquette state looking like on Supplementary Fig. 1. The energy of this configuration is that of a loop cut, i.e. ζ. Acting a second time with $H_{\rm kin}$, with the "active" bond operator one bond away from the first active bond creates another configuration of energy ζ. Only at third order is the system brought back to the loop manifold. There are twelve ($= 6 \times 2$) ways to achieve this. It is noteworthy that including many other terms from Supplementary Eq. (1) will not produce a lower-order contribution.

Supplementary Note 3. Haldane chain energy

In this appendix we investigate the energy density (energy divided by the number of sites) of $S = 1$ loops in the Haldane phase as a function of their length.

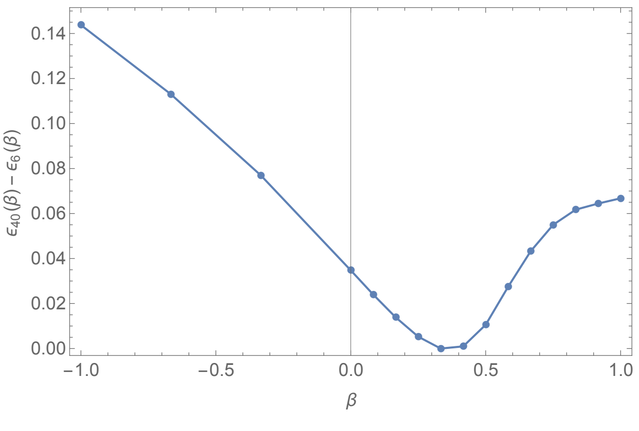

Supplementary Fig. 2. **Difference in ground state energy density of periodic chains of length 40 and 6**, as a function of β, as calculated in DMRG, in units of J. The energy density at length 40 is "assumed" to be close to that of infinite-length chains.

AKLT chains.— At the AKLT point, the energy density is independent of the loop length. Indeed the AKLT Hamiltonian may be rewritten as

$$H_{\rm AKLT} = \frac{1}{4} \sum_i \left[(\mathbf{S}_i + \mathbf{S}_{i+1})^2 \left((\mathbf{S}_i + \mathbf{S}_{i+1})^2 - 2 \right) + {\rm const} \right], \qquad (5)$$

i.e. as the sum of the projectors (with equal *positive* coefficient) onto the $S^{\rm tot} = 2$ sector (i.e. $(\mathbf{S}^{\rm tot})^2 = 2(2+1) = 6$) of the $\mathbf{S}_i^{\rm tot} = \mathbf{S}_i + \mathbf{S}_{i+1}$ operator. This means that the ground state will have zero components in the $S = 2$ sector. Then, the energy is independent of chain length. Hence, at first order in perturbation theory, the spins do not lift the degeneracy of the loop coverings at the spin AKLT point.

Numerical results away from the AKLT point.— We performed exact diagonalization for the Hamiltonian in Eq. (1) of the main text on chains with periodic boundary conditions for up to length 7. The results seem to indicate that, away from the AKLT point $\beta = 1/3$, the energy density of *closed* even-length loops (relevant for the honeycomb and hyperhoneycomb lattices) increases with loop length. Results obtained in DMRG for longer closed loops with the use of the itensor package confirm that the energy density of loops of length 40 is always larger than that of length 6, see Supplementary Fig. 2 (and Supplementary Ref. 3 for Monte Carlo results at $\beta = 0$).

β	-1	$-2/3$	$-1/3$	0	$1/12$
E_6^0	-24.8774	-19.2953	-13.8305	-8.61742	-7.38496
E_6^1	-22.6517	-17.6728	-12.7378	-7.8968	-6.71459
$\breve{E}_6^0$	-21.8484	-16.901	-12.0325	-7.37027	-6.2737
β	$1/6$	$1/4$	$1/3$	$5/12$	$1/2$
E_6^0	-6.19548	-5.06111	-4	-3.03786	-2.1977
E_6^1	-5.55072	-4.41121	-3.30427	-2.24068	-1.27149
$\breve{E}_6^0$	-5.22356	-4.2353	$-10/3$	-2.55428	-1.91296
β	$7/12$	$2/3$	$3/4$	$5/6$	$11/12$
E_6^0	-1.47434	-0.834305	-0.244138	0.317211	0.861294
E_6^1	-0.543207	0.163247	0.848938	1.51519	2.16354
$\breve{E}_6^0$	-1.36832	-0.874155	-0.406012	0.0474453	0.491835
β	1				
E_6^0	1.39445				
E_6^1	2.7956				
$\breve{E}_6^0$	0.930216				

Supplementary Table 1. **Ground and excited state energies of length-6 Haldane chains for different values of β and boundary conditions.** Ground state energy, first excited state energy of periodic Haldane chains (E_6^0 and E_6^1, respectively) and ground state energy of open Haldane chains ($\breve{E}_6^0$) of length six as a function of β (obtained in exact diagonalization), in units of J.

[1] Giniyat Khaliullin, "Orbital order and fluctuations in Mott insulators," Progress of Theoretical Physics Supplement **160**, 155–202 (2005).
[2] Giniyat Khaliullin, "Excitonic magnetism in van Vleck-type d^4 Mott insulators," Phys. Rev. Lett. **111**, 197201 (2013).
[3] Gang Sun, "Numerical solution of the spin-1 Heisenberg antiferromagnetic chains by using a projector method," Communications in Theoretical Physics **18**, 137 (1992).
