# Supplementary material for: Quantum loop states in spin-orbital models on the honeycomb lattice
Source: Nat Commun. 2021 May 21;12:3004. doi: 10.1038/s41467-021-23033-y (PMC8139991; doi:10.1038/s41467-021-23033-y)
Supplement: Supplementary file 2 — Peer Review File [file 41467_2021_23033_MOESM2_ESM.pdf]

Editorial Note: This manuscript has been previously reviewed at another journal that is not operating a transparent peer review scheme. The manuscript was considered suitable for publication without further review at Nature Communications .
